# Effects of Cytochrome P450 Inhibitors on Itraconazole and Fluconazole Induced Cytotoxicity in Hepatocytes

**DOI:** 10.1155/2009/912320

**Published:** 2009-07-16

**Authors:** Nhareet Somchit, Chong Sock Ngee, Azhar Yaakob, Zuraini Ahmad, Zainul Amiruddin Zakaria

**Affiliations:** ^1^Pharmacology and Toxicology Unit, Department of Biomedical Sciences, Faculty of Medicine and Health Sciences, University of Putra Malaysia (UPM), Serdang, 43400 Selangor, Malaysia; ^2^Faculty of Pharmacy, MARA University of Technology, 40500 Shah Alam, Malaysia

## Abstract

Itraconazole and fluconazole have been reported to induce hepatotoxicity in patients. The present study was designed to investigate the role of cytochrome P450 inhibitors, SKF 525A, and curcumin pretreatment on the cytotoxicity of antifungal drugs fluconazole and itraconazole. For 3 consecutive days, female rats were administered daily SKF 525A or curcumin (5 and 25 mg/kg). Control rats received an equivalent amount of dosed vehicle. The animals were anaesthetized 24 hours after receiving the last dose for liver perfusion. Hepatocytes were then exposed to various concentrations of antifungal drugs. In vitro incubation of hepatocytes with itraconazole revealed significantly lower viability when compared to fluconazole as assessed by lactate dehydrogenase, aspartate aminotransferase and alanine aminotransferase activities. The cytotoxicity of itraconazole was enhanced when incubated with hepatocytes pretreated with SKF 525A. SKF 525A had no effects on the cytotoxicity of fluconazole. Curcumin failed to either increase or decrease the cytotoxicity of both antifungal drugs. ATP levels also showed significant decrease in both itraconazole and fluconazole incubated hepatocytes. However, SKF 525A pretreated hepatocytes had significantly lower ATP levels after itraconazole incubations. Collectively, these results confirm the involvement of cytochrome P450 in the cytoprotection in itraconazole induced hepatocyte toxicity. Differences of the effects of SKF 525A on the cytotoxicity induced by itraconazole and fluconazole may be due to the differences on the metabolism of each antifungal drug in vivo.

## 1. Introduction

**Figure 1:**
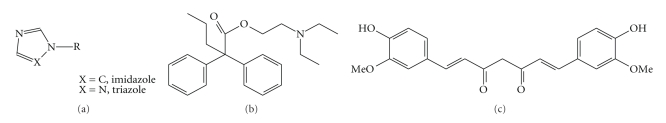
Chemical structures. (a) Azole ring. (b) SFK 525A (Proadifen). (c) Curcumin.

Itraconazole and fluconazole are triazole antifungal drugs, which are multiringed synthetic compounds containing three nitrogen atoms in the azole ring (Figure 1). The triazole drugs are broad-spectrum antifungal agents and are currently used to treat infections caused by various pathogenic yeast and molds [1]. The drugs are shown to be effective in both animal models [2, 3] and clinically [4, 5]. Mechanistically, the drugs inhibit the synthesis of ergosterol, which is an essential component of fungal cell membranes causing abnormalities in the membrane permeability, causing death to the cell [6].

The triazoles are thought to have greater antifungal potency, lower toxicity, and a wider antifungal spectrum than the older imidazole (Figure 1) antifungal drugs [1, 7]. However, there have been reports that itraconazole and fluconazole induced adverse drug reactions (ADRs). These include mild reversible ADRs such as gastrointestinal disturbances (dyspepsia, nausea, abdominal pain, and constipation), dizziness, and pruritis. Rare but severe hepatotoxicity has also been reported in patients undergoing itraconazole or fluconazole therapy [8, 9]. The mechanism of triazole-induced liver damage is unknown. Previous studies had suggested that the hepatotoxicity could be due to metabolic idiosyncrasy [9, 10]. 

Curcumin (difeuryloylmethane, Figure 1), the yellow colour of turmeric (*Curcuma longa*), a common ingredient in Asian cookery and traditional medicinal mixtures possesses a wide range of pharmacological properties that include antiinflammatory, antioxidant, and anticancer effects [11]. Curcumin is known to protect liver against the toxic effects of agents such as galactosamine, carbon tetrachloride, and acetaminophen [12]. There is evidence that curcumin enhanced liver detoxification activity and removal of toxic metabolites from the body [13]. Thapliyal and Maru [14] reported curcumins inhibited the activity of cytochrome P450 in vitro and in vivo.

Previously, we reported phenobarbital inhibited the in vitro cytotoxicity of itraconazole but not fluconazole in rat hepatocytes [15]. It was concluded that cytochrome P450 may be involved in the metabolism of itraconazole in the liver to remove the toxic metabolite/s and may not be involved in the metabolism of fluconazole. Recently, phenobarbital has been shown to reduce the hepatotoxicity induced by itraconazole in vivo [16, 17]. Inhibition of cytochrome P450 may increase the hepatotoxicity induced by itraconazole. In vitro techniques have been used extensively for investigations into hepatotoxicity of drugs and their metabolites [15, 18]. Therefore, the objective of this present study is to evaluate the effects of cytochrome P450 inhibitors, SKF 525A, and curcumin (Figure 1) on the in vitro cytotoxicity of itraconazole and fluconazole in rat hepatocytes.

## 2. Materials and Methods

### 2.1. Materials

The compounds were obtained from following sources: itraconazole was purchased from Janssen-Cilag (US) and fluconazole from Pfizer (France). Curcumin was obtained from ChromaDex (US). Proadifen (SKF 525A), dimethyl sulfoxide (DMSO), tricaprylin, Phenobarbital sodium, Leibovitz Glutamax I medium (L-15), Hank's balanced salt solution (HBSS), collagenase A (0.5 units/mg), hydrocortisone-21-sodium succinate, insulin, gentamycin, trypan blue (0.4% solution), cell culture water, bovine serum albumin (fraction V) and foetal calf serum were purchased from Sigma Chemical (UK). Sterile culture plates (60 and 90 mm diameter) and 50-mL culture flasks were purchased from Nunclon1 (Nunc, UK). All other chemicals are of the highest grade commercially available from Sigma Chemicals or Aldrich Chemicals, UK.

### 2.2. Animals and Isolation of Hepatocytes

Female Sprague-Dawley rats (200–250 g in body weight, *n* = 6/group) were housed in plastic cages with wood shavings as bedding. The rats were fed on rat pellets and tap water ad libitum. The care and experimental procedures were carried out in strict compliance with the Animal Ethics Committee rules and regulation followed in this institute. SKF 525A (5 and 25 mg/kg in saline) and curcumin (5 and 25 mg/kg in corn oil) were injected intraperitoneally for 3 consecutive days. Control animals received either saline or corn oil for 3 days. The animals were anaesthetized using pentobarbitone sodium at 60 mg/kg ip 24 hours after receiving the last dose for liver perfusion.

Hepatocytes were isolated by a two-step collagenase perfusion technique as previously described [15, 18, 19]. After isolation, hepatocyte suspensions were incubated at a density of 1 × 10^6^ viable cells/mL in L15 medium. Itraconazole or fluconazole (0.001, 0.01, 0.1, and 1.0 mM) were added in DMSO (final DMSO concentration of 1.0% v/v). Control hepatocyte suspensions were incubated with an equivalent amount of DMSO. The flasks were sealed in 95% O_2_/5% CO_2_ and placed in a shaking water bath at 37°C. Samples were taken from these flasks at time points of 0, 0.5, 1, 2, 3, 4, 5, and 6 hours.

### 2.3. Evaluation of Azole-Induced Cytotoxicity

Cytotoxicity was quantitatively assessed by measurement of lactate dehydrogenase (LDH, EC 1.1.1.27) release from hepatocytes into medium spectrophotometrically as described by Marshall and Caldwell [20]. For each time point, cytotoxicity was expressed as LDH activity in the medium as a percentage of total LDH activity in that flask (activity of LDH in the medium plus activity released by viable cells lysed by Triton X-100).

Enzyme activities of aspartate aminotransferase (AST, EC 2.6.1.1) and alanine aminotransferase (ALT, EC 2.6.1.2) were assayed after 6 hours incubations. AST and ALT activities were assayed using commercial test kits from Sigma Chemicals.

### 2.4. ATP Measurement

Separate incubations were performed for ATP determination using a commercial ATP assay system kit (Promega). Detailed methods as previously described by Qian et al. [21].

### 2.5. Statistical Analysis

Data was expressed as mean + SD and analysed using student's *t* test or Analysis of Variance (ANOVA). When interactions were significant, Duncan multiple posttest was performed. Values of *P* ≤ .05 was considered significant.

## 3. Results

**Figure 2:**
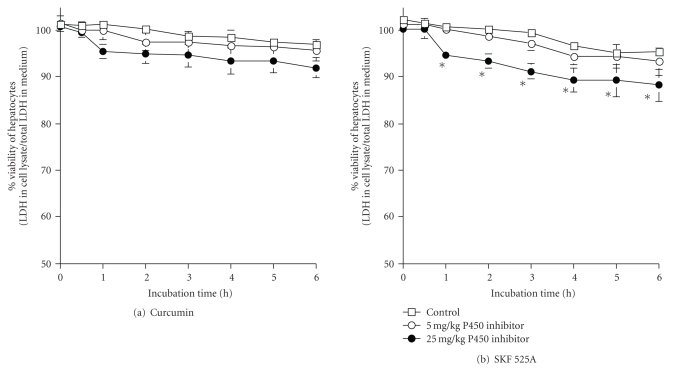
Effects of Cytochrome P450 inhibitors on the percentage viability of hepatocytes. *Significant different (*P* < .05) when compared to controls. Values are mean ± sd of 3 separate experiments.

Figures 2(a) and 2(b) illustrate the effects of cytochrome P450 inhibitors, SKF 525A, and curcumin to the viability of hepatocytes. Significant reduction in cell viability was observed in hepatocytes pretreated with 25 mg/kg SKF 525A only after 1 hour incubation. Curcumin at 25 mg/kg body weight reduced the viability of hepatocytes only after 6 hours incubation. No significant changes were detected in cell viability for both SKF 525A and curcumin at 5 mg/kg body weight.

**Figure 3:**
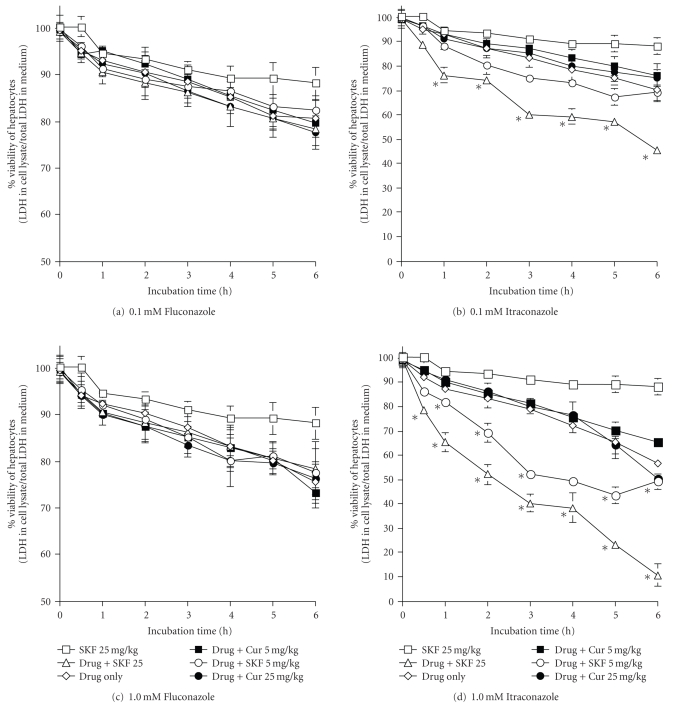
Effects of Cytochrome P450 inhibitors on the viability of hepatocytes treated with fluconazole or itraconazole. Values are mean ± sd of 3 separate experiments. *Significant different (*P* < .05) when compared to controls and drug only groups. Drug = itraconazole or fluconazole; Cur = curcumin; SKF = SKF 525A.

The cytotoxicity of fluconazole and itraconazole was observed to be time- and concentration-dependent (Figures 3(a) to 3(d)). Itraconazole induced statistically significant LDH leakage at 0.01 mM (Figure 3(b)), whereas fluconazole was cytotoxic at 1.0 mM (Figure 3(c)). At the highest drug concentration, itraconazole incubated hepatocytes were approximately 58% viable compared to 77% for 1.0 mM fluconazole (Figures 3(c) and 3(d)). At concentration of 0.001 and 0.01 mM, both drugs were less toxic to hepatocytes (Data not shown).

Curcumin pretreatment at 5 and 25 mg/kg revealed no effects on the cytotoxicity of both fluconazole and itraconazole. Interestingly, pretreatment of hepatocytes with 5 and 25 mg/kg SKF 525A significantly enhanced the cytotoxicity of itraconazole, which can be clearly seen at 0.1 and 1.0 mM drug concentration (Figures 3(b) and 3(d)). The cytotoxicity effect was expressed in a dose dependent manner. The pretreated hepatocytes were statistically less viable than the non-pretreated cells immediately after 30 minutes incubation with 0.1 mM itraconazole. Non-pretreated hepatocytes and SKF 525A pretreated incubated with 0.1 mM itraconazole for 6 hours had viability of approximately 45 and 74%, respectively (Figure 3(b)). The viable hepatocytes were approximately only 9.5% after 6 hours incubation with 1.0 mM itraconazole (Figure 3(d)). SKF 525A had no effect on the cytotoxicity of fluconazole.

**Figure 4:**
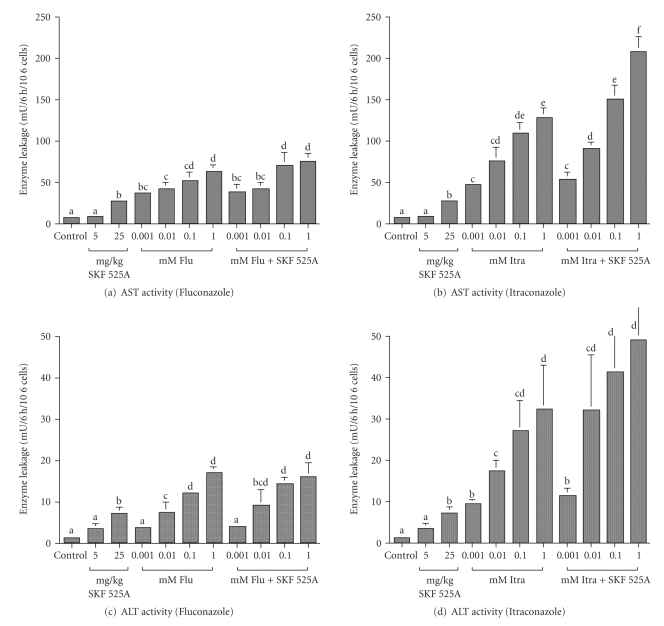
AST and ALT activities in rat hepatocytes exposed to various concentrations of itraconazole and fluconazole. ^a–d^Means with different superscript differ significantly (*P* < .05). Values are mean ± sd of 3 separate experiments.

 Similar trends were also observed in AST and ALT activities. Pretreatment with SKF525A enhanced the release of AST and ALT enzymes into the medium in itraconazole incubated hepatocytes (Figures 4(b) and 4(d)). Fluconazole only induced a marginal increase of both these enzymes activities (Figures 4(a) and 4(c)). Exposure to the antifungal drugs also caused concentration-related decrease in ATP was observed. Itraconazole incubated hepatocytes showed lower ATP levels when compared to fluconazole (Table 1). It is interesting to be observed after SKF 525A pretreatment, ATP levels of hepatocytes incubated with itraconazole were even lower (Table 1). SKF 525A had no effects on the lowering of ATP levels in fluconazole incubated hepatocytes.

## 4. Discussion

The data presented in this current investigation reflect the utilization of in vitro model and cytochrome P450 inhibitors in evaluating the mechanism of cytotoxicity of antifungal drugs fluconazole and itraconazole. As our previous study [15], itraconazole was more cytotoxic to hepatocytes than fluconazole. This present study revealed the cytotoxicity of itraconazole was enhanced by a cytochrome P450 inhibitor SKF 525A as judged by assessing LDH, AST, and ALT activities. This inhibitor had no effect on the cytotoxicity of fluconazole. From a mechanistic perspective, cytochrome P450 plays a key role in the deactivation/detoxification of itraconazole or its metabolite/s. In addition, the hepatotoxcity of itraconazole also reduced hepatocytes ATP levels.

In vitro techniques are useful tools for investigating toxicity of drugs and their metabolites [22]. The function of cytochrome P450 in toxicity process was tested with P450 inhibitors (SKF 525A and curcumin). SKF 525A was slightly toxic to hepatocytes at 25 mg/kg pretreatment. Our previous study revealed that phenobarbital (an inducer of cytochrome P450) pretreatment prevented the cytotoxicity of itraconazole in vivo [17]. Based from our previous and current findings, we can strongly conclude that cytochrome P450 is involved in the detoxification of itraconazole or its reactive metabolite/s. Indeed, cytochrome P450 is responsible for the metabolism of itraconazole and many other azole in the liver [23]. However, we are still unsure of which metabolite/s or the parent drug itself is responsible for the hepatotoxicity observed clinically.

On the other hand, azole antifungal drugs have been demonstrated to inhibit cytochrome P450 [24]. Itraconazole has less inhibitive properties than the imidazoles in rat liver microsomes [25]. At higher doses however, it is possible that itraconazole can cause an autoinhibition of its metabolism and induced enhanced toxicity as observed in this current study. Even though fluconazole has been shown to be a potent cytochrome P450 inhibitor [26], this drug is excreted mainly unchanged/not metabolized [26, 27]. Cytochrome P450 may play a less role in the cytotoxicity of fluconazole. Inhibition of cytochrome P450 also may force an alternative route for metabolism of itraconazole. With metabolism via cytochrome P450 inhibited or impaired (by SKF 525A and/or auto-inhibition), this drug may then be metabolized by the flavin-containing monooxygenase (FMO). Rodriguez and Acosta [9] postulated FMO metabolism maybe responsible for ketoconazole-induced hepatotoxicity.

Curcumin had no effects on the cytotoxicity of itraconazole even though it has been proven to be a cytochrome P450 inhibitor in vivo and in vitro [14]. The failure of curcumin to enhance itraconazole cytotoxicity may be due to its inhibition of different P450 isoenzymes or less potency in comparison to SKF 525A [28]. Thapliyal and Maru [14] demonstrated potent inhibition of cytochrome P450 at 1% curcumin w/w in diet, the current doses of curcumin used in this present study given IP may not achieved the effective dose. Furthermore, by giving curcumin IP, this may change the metabolism of curcumin that may reduce its potency. Curcumin however, plays an important role in the hepatoprotectant in acetaminophen, galactosamine, and carbon tetrachloride poisoning [12]. Glutathione has been implicated in the prevention of hepatotoxicity of these compounds. Most likely, curcumin is a better inducer of glutathione-related enzymes [13] than inhibitor of cytochrome P450 [14]. Thus, glutathione may not be an important detoxification pathway of itraconazole.

Results from this current study revealed ATP levels in hepatocytes reduced dose-dependently after incubation with both itraconazole and fluconazole. Itraconazole being more potent inhibitor and this was enhanced by pretreatment with SKF 525A. In contrast, triazole antifungal drugs (itraconazole and fluconazole) are shown to have less effect on the mitochondrial functions if compared to imidazole antifungals (ketoconazole and miconazole) [9]. Imidazoles potently inhibit NADH oxidase and succinate dehydrogenase. The drug concentration of 1.0 mM used in this study may be too high that caused disruption of the whole hepatocytes rather than inhibition of mitochondrial respiration.

The effect of itraconazole and fluconazole on the liver has been recorded in vivo in an animal model. Subchronic dosing of rats with itraconazole resulted in histological changes in the liver marked by necrosis, bile duct hyperplasia, and biliary cirrhosis [16]. These were accompanied by liver enlargement and significant increase in the liver enzyme activities (alanine aminotransferase/ALT and alkaline phosphatase/ALP). However, rats dosed with fluconazole only revealed mild degenerative changes in the centrilobular region of the livers with no changes in the liver enzyme activities. Clinically, several patients on chronic itraconazole therapy experienced elevated ALT and ALP activities [29]. Hepatotoxicity induced by itraconazole in patients although rare, are more frequent when compared to fluconazole [1]. The mechanism underlying the hepatotoxicity remains unclear but cytochrome P450 may play a key role in preventing certain azole induced liver toxicity especially itraconazole. Based on the current findings and our previous studies, it can be strongly suggested that the hepatotoxicity could be due to metabolic idiosyncracy. In summary, itraconazole is more cytotoxic than fluconazole in vitro and cytochrome P450 is involved in the detoxification of itraconazole but not fluconazole.

## Figures and Tables

**Table 1:** ATP levels in rats hepatocytes after fluconazole or itraconazole incubations. Control and SKF 525A pretreated hepatocytes were exposed to various concentrations of fluconazole or itraconazole for 6 hours.

	ATP (nmol/10^6^ cells)		
Drug	Control hepatocytes	5 mg/kg SKF 525A pretreated hepatocytes	25 mg/kg SKF 525A pretreated hepatocytes
None (control)	27.1 ± 3.5^ax^	25.2 ± 2.7^ax^	23.2 ± 2.9^ax^
Fluconazole (mM)			
0.001	30.1 ± 4.5^ax^	27.5 ± 3.4^ax^	26.2 ± 4.1^ax^
0.01	31.2 ± 2.3^ax^	27.2 ± 2.1^ax^	25.1 ± 2.3^ax^
0.1	24.6 ± 0.7^bx^	24.0 ± 1.7^ax^	20.2 ± 5.2^ax^
1.0	20.3 ± 3.9^bcx^	20.1 ± 2.4^bx^	18.5 ± 6.7^ax^
Itraconazole (mM)			
0.001	29.2 ± 3.1^ax^	26.2 ± 3.1^axy^	21.2 ± 3.9^ay^
0.01	22.7 ± 2.4^bx^	19.3 ± 2.8^bcy^	16.2 ± 2.5^by^
0.1	16.3 ± 3.5^cdx^	15.7 ± 3.9^cdx^	12.3 ± 2.0^by^
1.0	13.2 ± 0.9^dx^	11.2 ± 2.9^dx^	7.2 ± 3.1^cy^

Values are mean ± sd of 3 separate experiments.

^a–d^Means with different superscript differ significantly (*P* < .05) in the same column.

^x-y^Means with different superscript differ significantly (*P* < .05) in the same row.
